# Plexiform Neurofibroma of the Posterior Tibial Nerve Misdiagnosed as Proximal Tarsal Tunnel Syndrome: A Case Report

**DOI:** 10.1055/s-0038-1632405

**Published:** 2018-02-28

**Authors:** Sang Hyun Nam, Jung Yeon Kim, Jaeki Ahn, Yongbum Park

**Affiliations:** 1 Department of Plastic and Reconstructive Surgery, Sanggye Paik Hospital, Inje University College of Medicine, Seoul, South Korea; 2 Department of Pathology, Sanggye Paik Hospital, Inje University College of Medicine, Seoul, South Korea; 3 Department of Physical Medicine and Rehabilitation, Sanggye Paik Hospital, Inje University College of Medicine, Seoul, South Korea

**Keywords:** neurofibroma, tarsal tunnel syndrome, Tinel's sign

## Abstract

Plexiform neurofibromas of the foot are rare, benign tumors of the peripheral nerves. Diagnosis can be challenging if they present with symptoms mimicking other peripheral nerve pathologies. Tarsal tunnel syndrome is an entrapment syndrome of the entire tibial nerve behind the medial malleolus and under the flexor retinaculum. The clinical presentation typically includes posteromedial pain, positive Tinel's sign, and neurogenic signs, including both the sensation of numbness and the actual hypoesthesia and clawing of the toes.

Here, we report the case of a 59-year-old female patient with plexiform neurofibroma with symptoms similar to those of tarsal tunnel syndrome. The plexiform neurofibroma was surgically excised and the nerve function was partially preserved.

Plexiform neurofibroma (PNF) arises as a diffuse mass from the nerve trunk and leads to overgrowth of cutis and subcutis structures. 1 They are composed of Schwann cells, perineural cells, mast cells, and fibroblasts. 2 PNFs are locally invasive and often cause significant pain, deformity, and functional problems of the involved part of the body because of their mass effect. 3 PNFs affect 20 to 40% of individuals with neurofibromatosis type I and are characterized by their propensity for malignant degeneration, occurring in approximately 10% of cases. 1 4 PNFs of the foot are rare, and no case of associated bone involvement in the foot has been reported. 3 The diagnosis of PNF may be challenging if the tumor mass is not visible under the skin or if the symptoms mimic those of other peripheral nerve disorders. 2

Proximal tarsal tunnel syndrome (TTS) is typically caused by a space-occupying lesion, such as a ganglion, lipoma, neurilemmoma, or bony fragments from a calcaneal fracture. 5 Accurate diagnosis can be difficult because symptoms are similar to those associated with other lower limb conditions. 6

Here, we report the case of a 59-year-old patient in which a PNF of the left posterior tibial nerve without any neurofibromatosis symptoms was misdiagnosed as TTS and was only correctly identified after the development of additional symptoms.

## Case Report

**Fig. 1:**
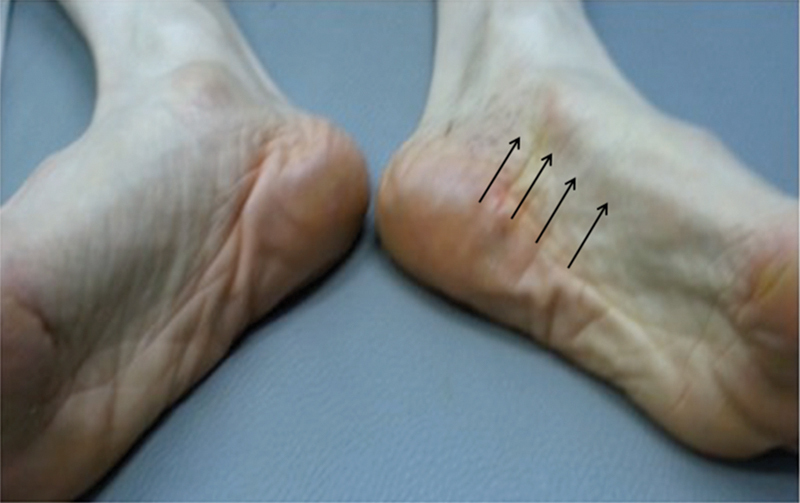
A 59-year-old woman presented with a 1-month history of numbness on the plantar surface of her left foot. Discomfort occasionally radiated proximally along the medial aspect of the calf. She had a traumatic history of hitting the bottom of her foot on a sharp rock. On physical examination, obvious wasting of the left abductor halluces and foot intrinsic muscles was seen (arrow).

**Fig. 2:**
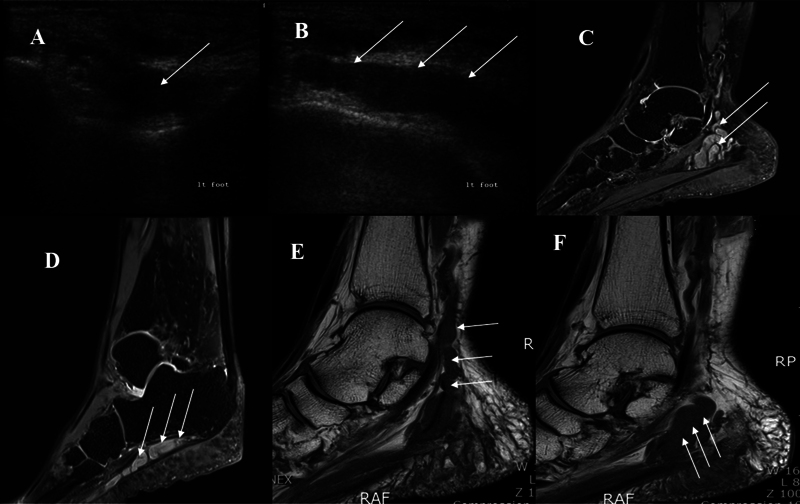
Plexiform neurofibroma. Multiple hypoechoic round masses surrounded by a hyperechoic background. The diameter of each mass measured 0.5 to 0.8 cm. ( **A** ) Short-axis ultrasound (US) image obtained over the calcaneus shows plexiform neurofibroma as a fusiform solid hypoechoic mass (arrow). ( **B** ) Long-axis US image obtained over the calcaneus shows plexiform neurofibroma as a cordlike hypoechoic mass (arrow). ( **C** ) T1-weighted image shows a multiloculated tubular lesion within the tarsal tunnel (arrow). ( **D** ) T1-weighted image shows a cordlike mass lesion below the calcaneus (arrow). ( **E** , **F** ) T2-weighted image shows a multiloculated tubulocystic lesion along the tibial nerve, lateral plantar nerve, proximal medial plantar nerve, and posterior calcaneal nerve, from the distal tibial plafond level to the fifth metatarsal base level.

A 59-year-old woman presented with a 1-month history of numbness on the plantar surface of her left foot. Discomfort occasionally radiated proximally along the medial aspect of the calf. She had a traumatic history of hitting the bottom of her foot on a sharp rock. On physical examination, obvious wasting of the abductor halluces and foot intrinsic muscles on the left foot was visible ( Fig. 1 ). Knee, ankle, and toe movements, and motor power were intact. On sensory examination, although the patient reported numbness, deep and peripheral senses in the extremity were completely normal, and no pathological reflexes were elicited. There were no subcutaneous fibromatoses or café au lait spots identified. A mass was palpable along the medial aspect of the ankle. Tinel's sign was positive in the posterior tibial nerve at the ankle. A nerve conduction velocity test revealed a smaller medial and plantar sensory action potential on the left side than on the right side, while compound muscle action potentials recorded from the left abductor hallucis and gastrocnemius showed smaller amplitudes than those on the right. Electromyogram studies showed denervation potential at the left abductor hallucis. Ultrasonography showed a heterogeneous, hypoechoic, and widely extending lesion. We also identified fusiform swelling of the nerve, which was indicative of tumor origin from a neurofibroma ( Fig. 2A , B ). Magnetic resonance imaging (MRI) showed a multiloculated tubulocystic lesion within the tarsal tunnel along the tibial nerve, lateral plantar nerve, proximal medial plantar nerve, and posterior calcaneal nerve, from the distal tibial plafond level to the fifth metatarsal base level. Additionally, atrophy of the posterior compartment muscles of the lower leg was observed, and this finding was more prominent especially in distal flexor hallucis longus, flexor digitorum longus, and lateral part of the soleus. Also, atrophy of the overall intrinsic muscles of the left foot was observed, especially in the interossei. The mass showed uniform low-signal intensity on T1-weighted images ( Fig. 2C , D ) and high-signal intensity on T2-weighted images ( Fig. 2E , F ). Findings on physical examination and imaging were consistent with a diagnosis of a PNF of the foot.

**Fig. 3:**
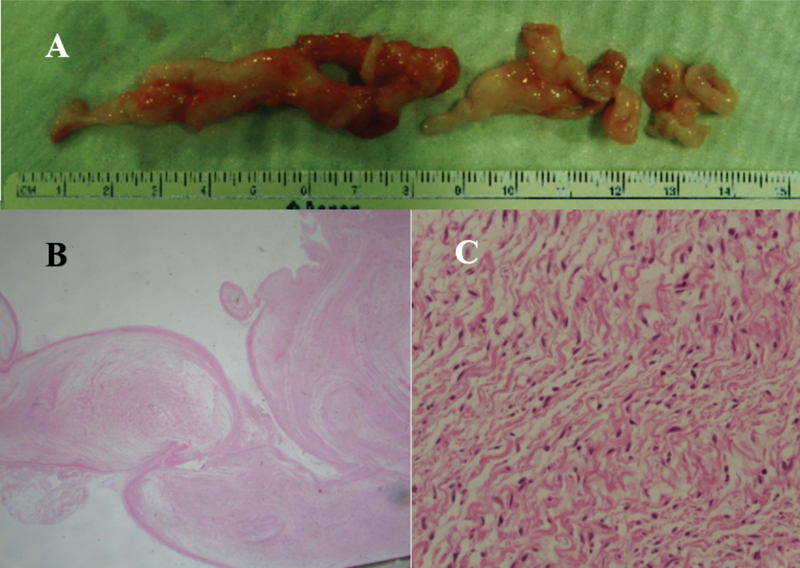
A multiloculated plexiform mass originated from the posterior tibial nerve ( **A** ). Plexiform arrangement (x10, hematoxylin and eosin [H&E] stain) ( **B** ) composed of wavy spindle cells (x400, H&E stain) ( **C** ) on histopathological examination.

The patient was referred to our foot surgery unit, where surgical excision of the lesion was performed. A curved skin incision was made over the tarsal tunnel. The flexor retinaculum was cut, and the underlying posterior tibial nerve and multilobulated plexiform mass were identified. The mass originated from the posterior tibial nerve and involved the calcaneal branch, medial plantar nerve, and lateral plantar nerve ( Fig. 3A ).

In the tarsal tunnel, nerve degeneration was confirmed by exploration along the course of the tibial nerve, and a plexiform mass was observed along the peripheral path of the nerve. After dissection, no cystic portion was observed. The mass was observed at the midfoot plantar aspect of the lateral plantar cutaneous nerve. The lesion artery and vein were compressed by the mass from the ankle to the midfoot transitional area, and decompression was sufficient. An **L** -shaped incision was made from 4 cm above the left foot medial malleolus to the talonavicular joint along the tibial nerve pathway. Soft tissues and the flexor retinaculum were removed and identified by tibial nerve exploration and by findings consistent with nerve degeneration. The posterolateral mass was identified by widening the operative site to the deep portion and then removing the mass from the surrounding tissues. In addition, after vertical incision of the midfoot lateral plantar aspect, the plantar first layer was dissected, the lateral plantar nerve on the quadratus plantae muscle was identified, and the mass was excised. Histopathological examination of the excised mass confirmed the diagnosis of PNF ( Fig. 3B , C ). The patient was referred for genetic screening, which was negative for neurofibromatosis type 1and type 2.

Postoperatively, she showed progressive improvement of foot function 3 months postsurgery. The strength of the intrinsic toe flexor and extensor muscles was normal. Neurologic examination revealed anesthesia of the medial aspect of the left sole; the pain completely disappeared, and she fully recovered to normal walking.

On ultrasonography performed 3 months after surgery, thickening of the tibial nerve with mild irregularity of the nerve fascicle and focal aging at the operative site (possibly due to postoperative scar change) was identified, and no definite evidence of a masslike lesion was visible at the operation site (tibial nerve branching site). In addition to these ultrasonographic findings, mild swelling of the tibial nerve, proximal to the surgical site, with three small hypoechoic nodular lesions within the nerve (2–3 mm), possibly representing small PNFs, was observed. There was no change in the size of the three small hypoechoic nodular lesions that had been previously seen on ultrasonography and no change in symptoms.

## Discussion

The remarkable features of our case are as follows: (1) erroneous interpretation of the patient's signs and symptoms as being caused by proximal TTS, (2) the occurrence of a PNF in a patient negative for genetic neurofibromatosis screening, and (3) the maintained ability of our patient to walk independently and correctly postsurgery, despite the partial removal of the posterior tibial nerve.

PNFs constitute a significant source of structural and functional morbidity in patients with neurofibromatosis type I. 7 PNFs are intimately associated with functional nerve fibers and fascicles, passing centrally within the tumor itself. As a result of their locally aggressive behavior, patients often present with significant pain, physical deformity, and progressive neurologic dysfunction secondary to tumor growth and compressive mass effect. 3 7

Any nerve can develop PNF; however, PNFs occur most commonly in the head and neck region due to the rich innervation of the area. PNF of the foot is uncommon. 8 Five cases have been reported in the literature. 8 The age of onset is variable since PNFs may develop throughout life and are usually present at birth. 7 They usually produce a slow-growing, well-circumscribed lesion in the peripheral nerve. They always present as a painless mass but less commonly with a neurologic deficit. 7 In our patient, the symptoms started 1 month prior to presentation, but the tumor developed long prior to this, as demonstrated by the degree of muscle atrophy in the distribution of the posterior tibial nerve.

TTS is the most common entrapment neuropathy of the lower limb. 5 6 The etiology of TTS includes trauma, heel varus, rheumatoid arthritis, and space-occupying lesions (ganglion, lipoma, accessory muscle, schwannomas, exostosis, varicosities). 5 6 9 TTS usually presents with sharp burning pain along the plantar aspect of the foot and medial ankle in association with paresthesia and numbness on the plantar foot. 9 Physical examination will show a positive Tinel's sign, wasting of the intrinsic muscles, and diminished foot sensation. 9 MRI is the gold standard for identification of suspected compression of the tarsal tunnel caused by the presence of obstructive foreign objects, lesions, or tumors. 6

In our patient, TTS was suspected based on physical examination, clinical symptoms, electromyography, and nerve conduction test. Ultrasonography and MRI were performed to confirm the diagnosis. The previous case reports have misdiagnosed PNF as an entrapment syndrome. D'Orazi et al report the case of a patient who had severe foot pain, which progressively hampered her walking ability and erroneously attributed to recurrent Morton's neuroma. 2 PNF of her right medial plantar nerve was diagnosed through an MRI. MRI demonstrated multinodular formations along the medial plantar nerve, isointense in T1-weighted and high-intense in T2-weighted images, hypothetically attributable to PNF or multiple neurinomas. She showed a progressive improvement of foot function 12 months after radical neurotomy of the medial plantar nerve. We suspected PNF based on all preoperative test results. After surgical treatment, PNF was confirmed by biopsy.

The basic histology of neurofibromas consists of small, bland, elongated spindle cells with characteristic wavy or comma-shaped nuclei arranged in a myxomatous stroma. 10 Definitive diagnosis is obtained by histopathological evaluation and, in some cases, immunohistochemical studies. PNF cells are usually S-100 positive, and in cases with less well-defined patterns or sparse cellularity, this characteristic staining pattern can be used to confirm the diagnosis. 10 The neurofibromatoses are autosomal dominant disorders, of which there are two distinct forms, type 1 and type 2. 2 The former is characterized by the presence of fusiform, globular, plexiform, or diffuse neurofibromas, whereas bilateral vestibular, cranial, spinal, and peripheral nerve schwannomas are the distinctive features of the latter. 2 The estimated risk of malignant peripheral nerve sheath tumors in patients with neurofibromatosis type 1 remains considerably high (∼5–13%, according to published series). 11 When the diagnosis of PNF was confirmed, our patient was referred for genetic screening of both neurofibromatosis type 1 and type 2, both of which were negative.

Although the treatment of PNF is still controversial, surgical excision is the only definite treatment. 12 The indications for surgical intervention include pain, dysfunction, diagnostic biopsy, and/or suspected malignancy. However, PNFs tend to recur in 20% of cases despite an appropriate surgical approach. 13

In our patient, neurologic examination showed a decrease in sensation on the medial side of the left foot sole, but her pain improved and she fully recovered to walk normally. Ultrasonography performed 3 months after the operation revealed no recurrence at the surgical site, and the symptoms did not recur.

## Conclusion

PNF is an uncommon nerve sheath tumor and is rarely identified in the extremities. We presented a case of PNF derived from the posterior tibial nerve with symptoms similar to those of TTS. Our patient was treated with surgical excision, and her symptoms improved dramatically. A correct diagnosis is essential prior to treatment of PNF since these tumors have the potential for malignant transformation, and misdiagnosis may lead to inappropriate treatment.
